# Corrigendum

**DOI:** 10.1155/DTE.4.107

**Published:** 1997


					Diagnostic and Therapeutic Endoscopy, Vol. 4, p. 107
Reprints available directly from the publisher
Photocopying permitted by license only
(C) 1997 OPA (Overseas Publishers Association)
Amsterdam B.V. Published under license
in The Netherlands under the Harwood Academic
Publishers imprint, part of The Gordon and
Breach Publishing Group.
Printed in Singapore.
CORRIGENDUM
In the paper entitled "Early Localization of Bronchogenic Cancerous/Precancerous Lesions with Lung
Imaging Fluorescence Endoscope" by N. Ikeda et al., published in Vol. 3 No. 4, pp. 197-201, the
illustration for Table II is incorrect. The correct table is now published below.
TABLE II Endoscopy and biopsy results
LIFE negative LIFE positive
Invasive cancer (13 sites)
BF negative 0
BF positive 0 12
Carcinoma in situ (3 sites)
BF negative 0 0
BF positive 0 3
Atypical squamous metaplasia (25 sites)
BF negative 0 18
BF positive 6
Normal/Inflammation (36 sites)
BF negative 11 11
BF positive 9 5
107

				

